# Characterization of energy filtering slit widths for MicroED data collection

**DOI:** 10.1016/j.yjsbx.2026.100158

**Published:** 2026-08-14

**Authors:** Max T.B. Clabbers, Johan Hattne, Michael W. Martynowycz, Tamir Gonen

**Affiliations:** a Howard Hughes Medical Institute, University of California, Los Angeles, CA 90095, United States; b Department of Biological Chemistry, University of California, Los Angeles, CA 90095, United States; c Department of Physiology, University of California, Los Angeles, CA 90095, United States

**Keywords:** cryoEM, MicroED, Microcrystal electron diffraction, Energy filter

## Abstract

A favorable signal-to-noise ratio is essential for obtaining high-quality diffraction data in macromolecular electron crystallography. Inelastic scattering contributes significantly to the noise, reducing contrast between diffraction peaks and background, which complicates peak detection and compromises the accuracy of intensity integration. Energy filtering mitigates these challenges and enhances diffraction data quality by removing inelastically scattered electrons, leading to reduced background noise and sharper Bragg peaks. Previously, we reported a substantial improvement in MicroED data quality and resolution with energy filtering. Here, we systematically evaluate the impact of different energy filter slit widths for optimal MicroED data collection. Data from proteinase K lamellae were collected using 5, 10, and 20 eV energy filter slits. Our results show that the optimized energy filtering reduces background counts and improves the signal-to-noise ratio, enhancing the precision of intensity measurements and resulting in improved structural models. These findings provide insight into the optimization of energy filter slit settings that, when paired with direct electron detection, enhance data collection strategies by improving the signal-to-noise ratio, supporting higher quality data, and ultimately enabling more precise structure determination.

## Highlights

• Systematic evaluation of energy filter slit widths for macromolecular MicroED experiments.
• A 5 eV energy filter slit provides the best overall MicroED data quality.
• Optimized energy filtering improves signal-to-noise ratio and structure refinement.

## Graphical abstract

**Figure:**
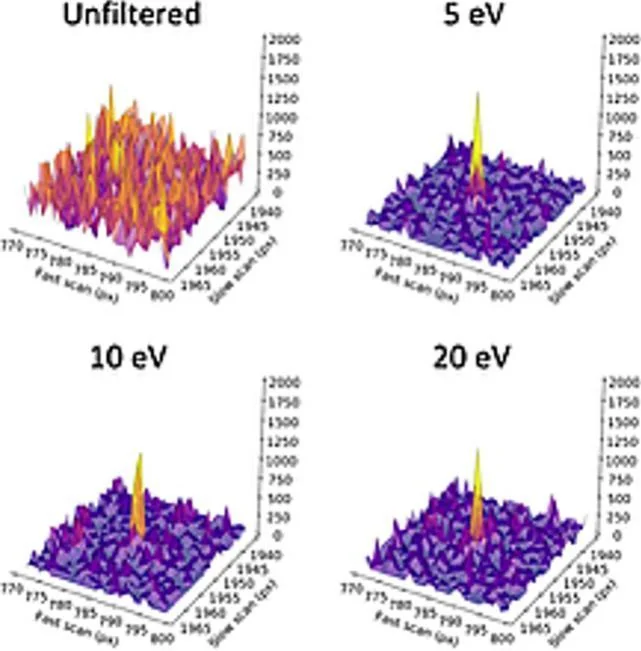

## 1 Introduction

Data collection is a critical part of any crystallographic experiment, where a higher signal-to-noise ratio (SNR) allows for more precise intensity measurements and ultimately leads to better structural models. Optimizing the experimental setup and data collection strategies is therefore essential to maximize the amount of useful structural information that can be extracted from each crystal. In X-ray diffraction, highly accurate data are collected at synchrotron facilities, however, radiation damage limits the minimum crystal size that can be used (Holton and Frankel, 2010; Sanishvili et al., 2011). In electron crystallography, the signal is generally much weaker because it relies on crystals that are much smaller, resulting in fewer repeating units to amplify the coherent kinematic diffraction signal. Fortunately, electrons deposit substantially less energy into the sample per useful elastic scattering event compared to X-rays (Henderson, 1995). This makes it possible to obtain sufficiently strong and highly accurate data from small crystals in microcrystal electron diffraction (MicroED) (Nannenga et al., 2014). Faster and more sensitive detectors have allowed continuous rotation data collection, and significantly improved data quality and resolution (Clabbers et al., 2017; Nannenga et al., 2014; Yonekura et al., 2015). More recently, we demonstrated that employing electron counting data collection on direct electron detectors further improves the SNR (Clabbers et al., 2022; Martynowycz et al., 2022). Even under low flux density conditions, these cameras are sensitive enough to capture the diffraction signal. Despite these advances, uncertainties in the diffraction geometry and sources of experimental noise persist, suggesting room for further optimization of hardware setups and data collection strategies to achieve higher resolution and improved data quality.

A major source of noise in electron microscopy is inelastic scattering, which can obscure otherwise valuable structural information. Inelastically scattered electrons lose a small fraction of their energy and undergo a phase shift, rendering them incoherent with the primary elastic scattering events (Cowley and Moodie, 1957). Inelastic scattering events therefore interfere with the coherent kinematic diffraction signal that is focused near the Bragg spots. This presents a significant challenge, as inelastic scattering occurs 3–4 times more frequently than elastic scattering in biological specimens (Henderson, 1995; Latychevskaia and Abrahams, 2019). Most inelastically scattered electrons will end up near the direct beam, as the scattering angle for inelastic events is generally relatively small. This leads to high background noise surrounding the beam center, which obscures the low-resolution diffraction spots. This inelastic background noise then rapidly falls off with increasing distance from the center. Additionally, there is a high probability of multiple scattering interactions due to the short inelastic mean-free-path of electrons (Fujiwara, 1959; Latychevskaia and Abrahams, 2019). Multiple scattering events involving an inelastic scattering component can contribute to the background noise and peak broadening. Electrons undergoing an elastic event followed by inelastic scattering become partially incoherent but remain distributed near the reciprocal lattice positions, contributing to the noise immediately surrounding the Bragg peaks (Latychevskaia and Abrahams, 2019). Both the increased background and peak broadening can interfere with data integration, affecting estimation of the intensities of the peak profiles, and complicating the detection of faint high-angle reflections that are at or below the noise level. To make matters worse, these effects can become even more pronounced as the path length the electrons traverse increases, either because of thicker samples or when measuring at higher tilt angles.

In electron microscopy, energy filtering can selectively remove inelastic electrons that have lost more than a specific amount of energy, effectively isolating the coherent elastic signal and improving the SNR. Previous reports assessed the improvements of both in- and post-column energy filters in macromolecular crystallography (Yonekura et al., 2002, Yonekura et al., 2015, Yonekura et al., 2019, Gonen, 2005, Gonen, 2004) and materials science (Gemmi and Oleynikov, 2013; Yang et al., 2022). These studies consistently demonstrate a clear reduction in background noise, sharper Bragg peaks, and improved intensity and model statistics. Recently, we demonstrated that combining energy filtering with direct electron detection significantly improves MicroED data quality and resolution compared to unfiltered data (Clabbers et al., 2025). In that study, a data collection strategy combining multiple small sweeps was implemented, to accommodate both the flux density limitations imposed by the detector in counting mode and the total fluence tolerated by the crystals. This strategy improved the resolution of proteinase K lamellae from 1.40 Å for unfiltered data to 1.09 Å resolution for filtered MicroED data (Clabbers et al., 2025). Importantly, this enabled the visualization of detailed structural features, including individual hydrogen atom positions in the higher-quality energy-filtered data. Furthermore, it was illustrated that high-resolution information, lost due to noise introduced by inelastic scattering, could be recovered by inserting the energy filter slit, even after the sample had already been pre-exposed (Clabbers and Gonen, 2025). While these studies focus on the comparison between filtered and unfiltered data, they did not examine how the choice of slit width itself potentially influences data quality. Although narrower slits are expected to reject a larger fraction of inelastically scattered electrons and therefore reduce background noise more efficiently, they may also attenuate the transmitted electron beam, limit the effective field of view, or become increasingly sensitive to instrument alignment. In particular, misalignment of the beam with respect to the apertures can exclude useful diffraction signal. Conversely, wider slits are more robust to alignment variations but allow more inelastic contributions to pass through. The optimal slit width is therefore not immediately obvious and understanding these effects is therefore critical in guiding practical experimental design and maximizing data quality.

Here, we systematically investigate this trade-off by comparing 5, 10, and 20 eV energy filter slit widths for macromolecular MicroED. We evaluate how slit width influences background suppression, diffraction signal contrast, intensity measurements, and structure refinement to identify conditions that maximize overall data quality.

## 2 Methods

### 2.1 Sample preparation

Crystals of proteinase K (*Engyodontium album*) were grown as described previously (Clabbers et al., 2025; Clabbers and Gonen, 2025; Martynowycz et al., 2022). The crystals were transferred onto an holey carbon electron microscopy (EM) grid (Quantifoil, Cu 200 mesh, R2/2) and vitrified using a standard deposit, blot, and vitrification protocol, described previously (Clabbers et al., 2025; Clabbers and Gonen, 2025). Lamellae of 300 nm thickness were prepared using a Helios Hydra 5 CX dual-beam plasma FIB/SEM (Thermo Fisher Scientific), following a milling protocol as outlined previously (Clabbers et al., 2025; Clabbers and Gonen, 2025). In each session, nine lamellae were prepared sequentially using the same protocol. Lamellae for the 5 and 10 eV slit setting tests were prepared from the same grid over two sessions. Lamellae for the 20 eV data were prepared from a second grid. After each session, grids were immediately cryo-transferred to the transmission electron microscope (TEM) for data collection.

### 2.2 Data collection

Diffraction data were collected on a Falcon 4i camera operated in electron counting mode using a Titan Krios G3i TEM (Thermo Fisher Scientific) that was set up for low-flux density diffraction, as described previously (Clabbers et al., 2025; Clabbers and Gonen, 2025). Diffraction stills were taken of a stationary crystal for brief 10 s exposures at a total fluence of ∼0.02 e^−^/Å^2^. Data were first collected from the same position without any energy filter slit inserted, followed by sequentially changing the slit width to 2, 5, 10, and 20 eV. MicroED datasets were collected using a strategy of multiple 20.0° sweeps, with longer 420 s exposures, a rotation speed of 0.0476°/s, and a total fluence of ∼0.84 e^−^/Å2, as described previously (Clabbers et al., 2025; Clabbers and Gonen, 2025). For each slit setting, data were collected from nine proteinase K lamellae. Frames were recorded in EER format and converted to SMV images using the MicroED tools (available at https://cryoem.ucla.edu/downloads). During conversion, the frames were binned by two in each direction and summed such that output images spanned 1 s, 2 s, or 3 s of rotation data, depending on the diffractive power of the sample.

### 2.3 Data processing and structure refinement

Data were processed using XDS (Kabsch, 2010). The resolution of individual datasets was truncated at the lowest *CC*_*1/2*_ that was still statistically significant (Karplus and Diederichs, 2012). For direct comparison of merged datasets collected with different slit widths, all data were merged and truncated at 1.2 Å resolution using XSCALE (Kabsch, 2010). At this resolution cutoff, the *CC*_*1/2*_ was approximately 30% in the highest resolution shell with a mean *I/σI* of 2.0 or higher. For each slit width setting, structures were refined using the same starting model (PDB ID 9DHO) (Clabbers et al., 2025) and refinement protocol in phenix.refine (Afonine et al., 2012)*,* including electron scattering factors, automated optimization of the geometry and ADP weights, and individual anisotropic *B*-factors. During refinement, no atoms were added to or subtracted from the models.

## 3 Results

### 3.1 Evaluation of energy filter slit widths on the diffraction signal

**Fig. 1:**
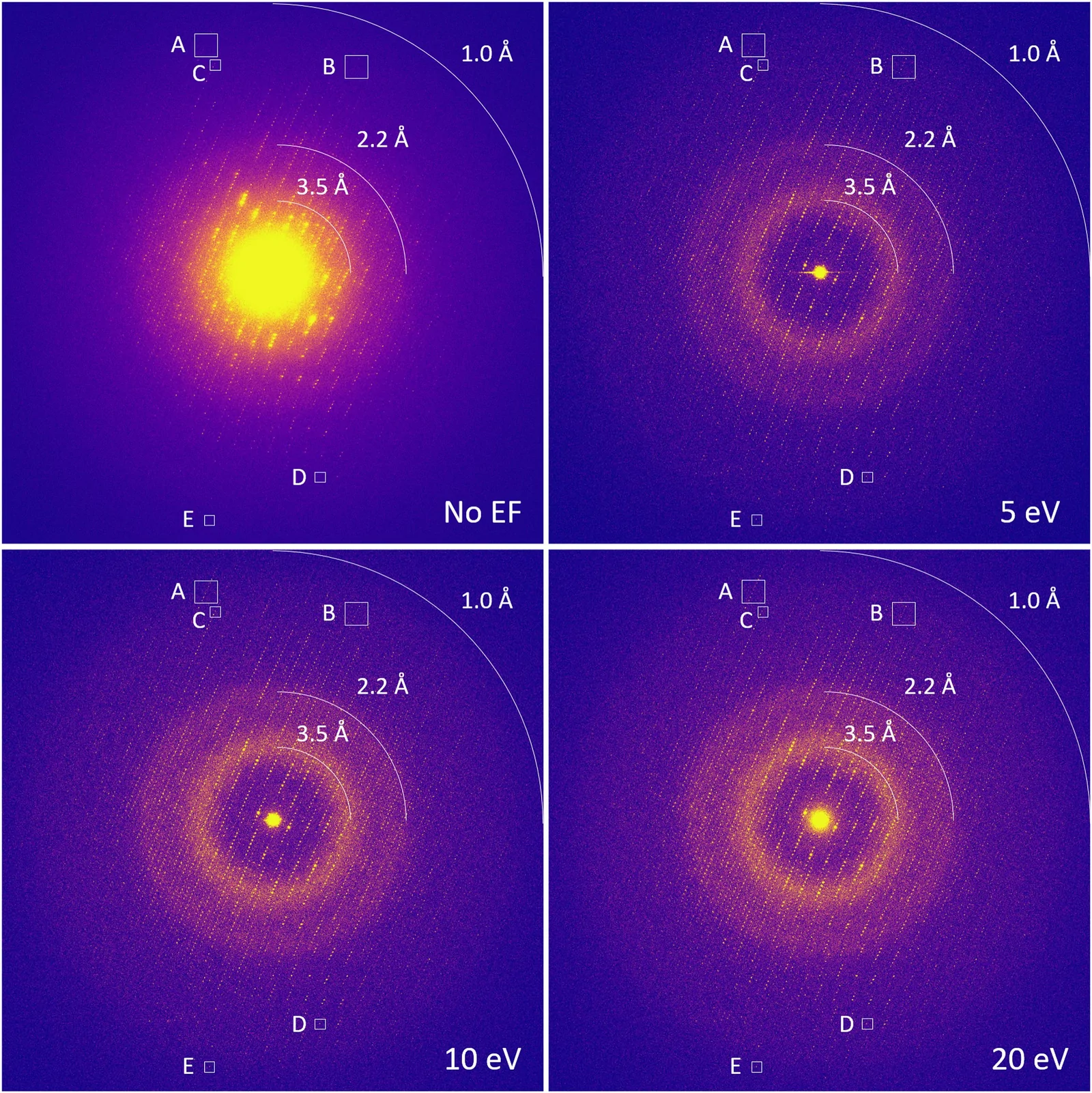
Filtered and unfiltered diffraction patterns of a proteinase K lamella taken first without the energy filter slit inserted, followed by the 5, 10, and 20 eV slit settings. Each diffraction pattern was taken at 10 s exposure with a total fluence of 0.02 e^−^/Å^2^. Diffraction patterns were taken at the same location on the lamella using a ∼ 3.5 μm parallel beam as defined by the SA aperture. The highlighted areas A-E were used to plot the peak profiles presented in Fig. 2.

To evaluate the different energy filter slit settings, diffraction patterns were recorded from the same area of a 300 nm thin proteinase K lamella that was kept stationary in the beam throughout. Each diffraction pattern was collected using a brief 10 s (∼3080 frames) exposure at a total fluence of 0.02 e^−^/Å^2^ by sequentially using no energy filter, followed by inserting the 5, 10, and 20 eV slit settings (Fig. 1). Each diffraction still was taken from the same location using a ∼ 3.5 μm diameter parallel electron beam as defined by the SA aperture. Visual inspection of the diffraction stills shows a substantial reduction in background noise and sharper peaks in all the filtered diffraction patterns compared to the unfiltered data. The better SNR is especially noticeable for the low-resolution reflections near the center beam position, which become clearly distinguishable from the background when the energy filter is used.

**Fig. 2:**
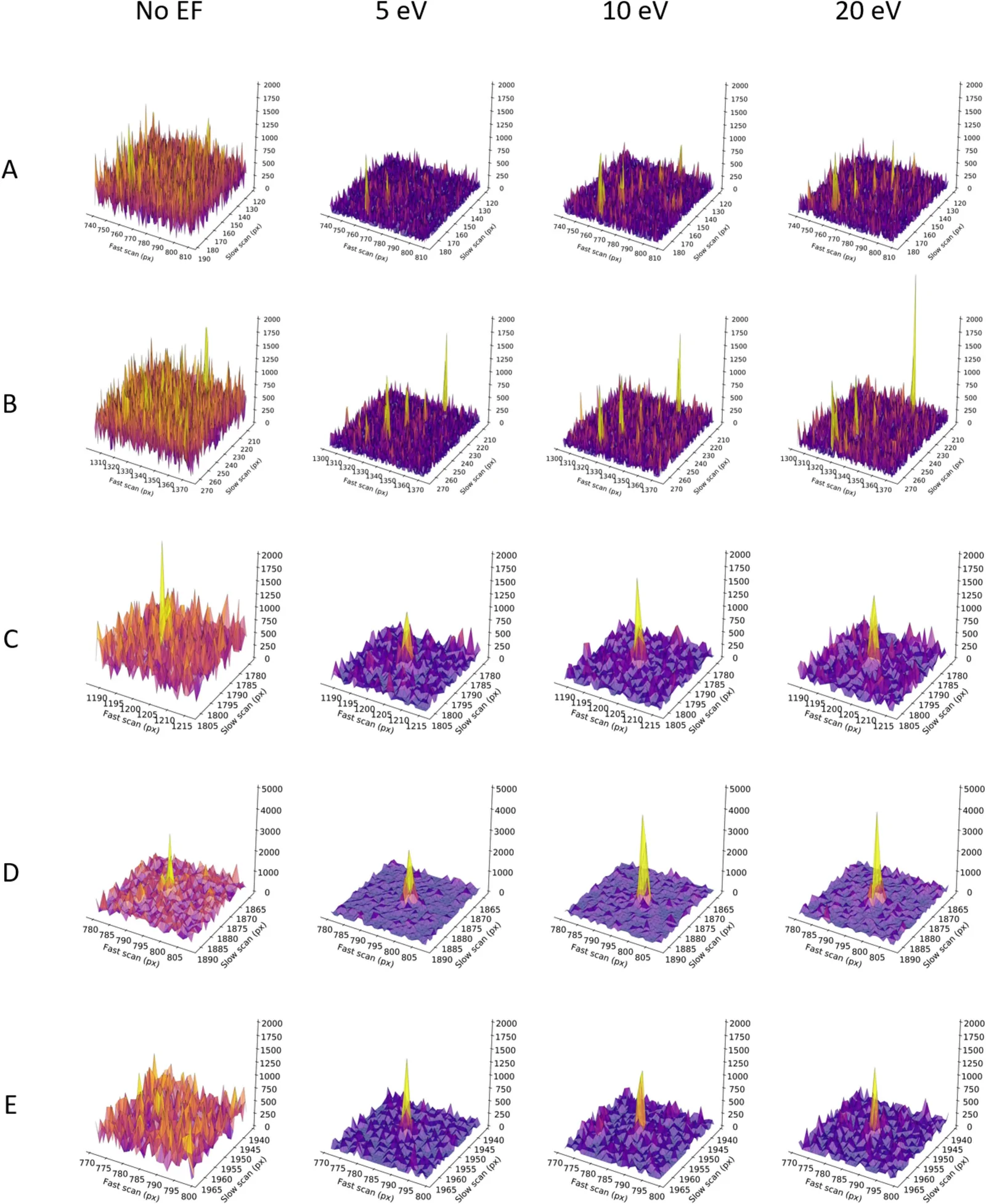
Peak profiles for unfiltered and filtered diffraction patterns taken from the same proteinase K lamella. Profiles are shown for the highlighted areas A-E in the diffraction data shown in Fig. 1. For reference, peak profiles are shown for Bragg spots at **A**: ∼1.07 Å resolution, **B**: ∼1.17 Å resolution, **C**: 1.25 Å resolution, **D**: 1.11 Å resolution, and **E**: 1.02 Å resolution.

With narrower energy filter slits, the background noise around the spots is reduced, improving the contrast of low-angle reflections and making spots at medium and high resolution clearer, extending the resolution of discernible diffraction to the edge of the detector at ∼1 Å resolution (Fig. 1). In comparison, any diffraction spots beyond approximately 1.2 Å resolution are difficult to separate from the background in the unfiltered data. Spot profiles shown at ∼1.2 and ∼ 1.1 Å resolution further illustrate this improvement, with rows of Bragg peaks appearing clearly above the noise level in unfiltered that were hidden within the much noisier background without energy filtering (Fig. 2A). Although filtered diffraction spots are generally less intense than unfiltered data, they nevertheless appear sharper due to the reduction of the background, as illustrated for individual spot profiles (Fig. 2B).

### 3.2 MicroED data collection at different energy filter slit widths

To further characterize the energy filter for optimal data collection, we compare intensity and model statistics for the 5, 10, and 20 eV slit widths. Electron counting requires low flux density conditions imposed by the count-rate capability of the camera, and a low total fluence imposed by the crystal (Hattne et al., 2023). This implies that the expected signal is proportionally weaker. Therefore, continuous rotation MicroED data were collected using an optimized strategy to maximize the SNR. This approach mitigates the above restrictions by rotating the crystals slower using an angular increment of 0.0476°/s and sampling small 20.0° sweeps in reciprocal space. The total fluence for each dataset was only ∼0.84 e^−^/Å^2^, and the absorbed dose by each crystal was equivalent to ∼3.08 MGy (Hattne et al., 2018). Nine crystals were used for MicroED data collection at each energy filter slit setting. For each slit setting, data were collected from nine lamellae to minimize variations between crystals and to ensure complete coverage of reciprocal space.

**Fig. 3:**
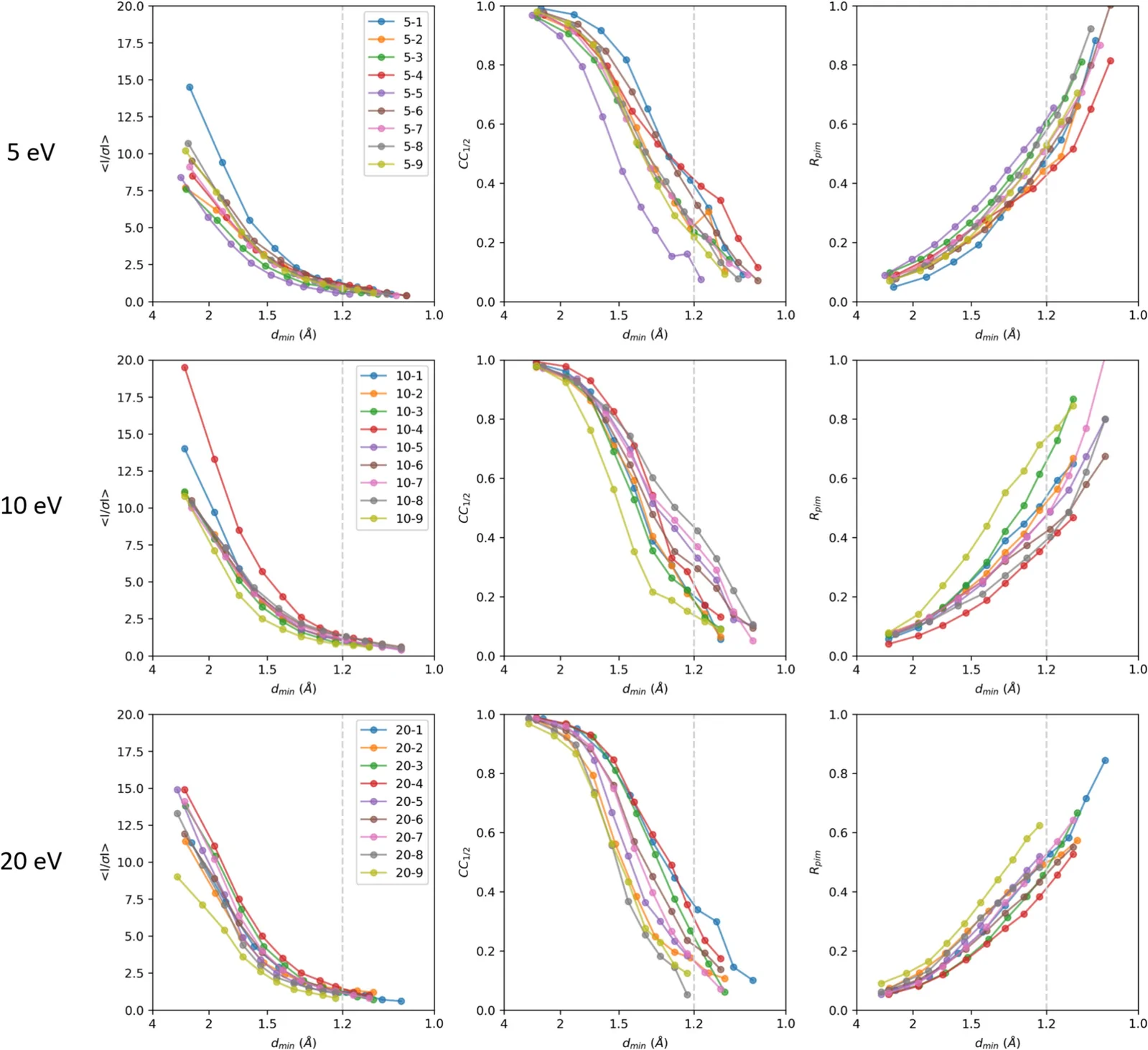
Intensity statistics for individual energy-filtered MicroED datasets collected using the 5, 10, and 20 eV slit settings. The crystallographic quality indicators mean *I/σI*, *CC*_1/2_, and *R*_pim_ are plotted as a function of the resolution. Individual datasets were truncated at a *CC*_1/2_ that was still significant at the 0.1% level in the highest resolution shell. For a side-by-side comparison, data for each slit width were merged and truncated at 1.2 Å resolution indicated by the grey line in each plot.

Overall, the filtered MicroED data show sharp and well-defined Bragg spots and low background noise. Individual datasets were processed and integrated up to a cross-correlation between two random half sets (*CC*_1/2_) that was still significant in the highest resolution shell (Karplus and Diederichs, 2012) (Fig. 3). All data showed strong diffraction, extending well beyond 1.2 Å resolution. Individual datasets collected with the 5 eV slit ranged between 1.18 and 1.05 Å resolution, the 10 eV data ranged from 1.13 to 1.06 Å resolution, and at 20 eV data were recorded from 1.22 to 1.06 Å resolution. Individual 10 eV datasets on average also showed higher resolution diffraction with four out of nine datasets having significant information at 1.06 Å resolution. Not all lamellae diffracted to the same resolution, which could be attributed to differences in crystallinity, quality of the lamellae, and specific lattice orientation. To quantitatively assess the reduction in background noise, radial intensity profiles were calculated from all diffraction patterns collected with each energy filter slit width (**Fig. S1**). The background intensity decreases markedly with energy filtering, contributing to the observed visual improvement in diffraction spot contrast. We also note that variability between datasets for a given slit width is greater than the difference between the average radial pixel value for each slit width.

**Fig. 4:**
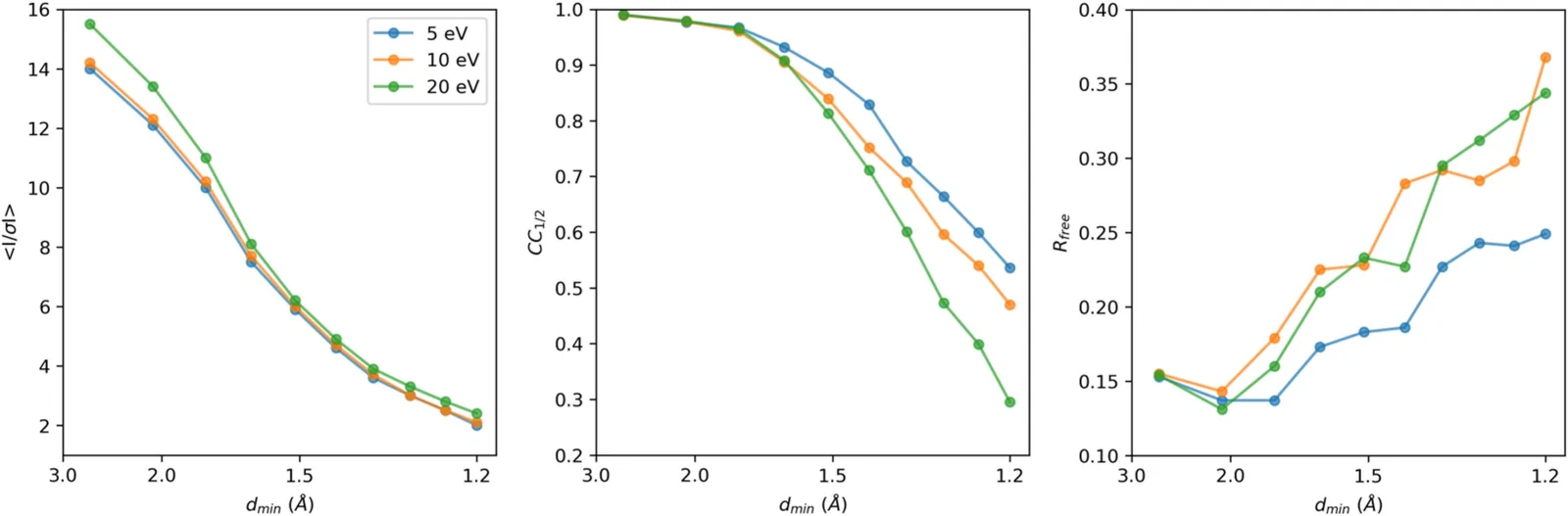
Intensity and model statistics for energy-filtered MicroED data collected using the 5, 10, and 20 eV slit widths. The mean *I/σI*, *CC*_1/2_, and *R*_free_ are plotted as a function of the resolution. For a side-by-side comparison, data for each of the slit settings were truncated at 1.2 Å resolution.

Data recorded at each energy filter slit width were merged and truncated at 1.2 Å resolution to enable a side-by-side comparison of the intensity and model statistics (Table 1**,** Fig. 4). At this resolution, each of the merged datasets had a *CC*_1/2_ of approximately 30% and mean *I/σI* > 2.0; the resolution for datasets collected with narrower slit widths would be higher if the high-resolution limit had been determined from data reduction statistics instead. Overall, the mean *I/σI* shows a slight increase, as well as in the highest resolution shell, going from the narrowest 5 eV slit to the 10 and 20 eV slit settings that let electrons with a greater energy loss pass the filter (Table 1). Yet overall, the mean *I/σI* follows a similar trend for all slit settings, except for the lower angle reflections of the 20 eV that are stronger (Fig. 4). The *CC*_1/2_ for the low-resolution reflections is similar between all slit settings, but then falls off rapidly for the 20 eV data to about 28.1%, whereas the 10 eV data fall off more slowly to 44.7%, and at 5 eV the falloff is even smaller with 50.5% in the highest resolution shell (Fig. 4). The data have similar multiplicity and show similar values for *R*_merge_.

**Table 1:** Data merging and refinement statistics.

	5 eV	10 eV	20 eV
**Data collection**			
No. of crystals	9	9	9
Space group	*P*4_3_2_1_2	*P*4_3_2_1_2	*P*4_3_2_1_2
Unit cell dimensions			
*a, b, c* (Å)	66.92, 66.92, 107.56	67.72, 67.72, 106.90	67.41, 67.41, 105.78
*α, β, γ* (°)	90, 90, 90	90, 90, 90	90, 90, 90
Resolution (Å)†	47.32–1.20 (1.23–1.20)	57.21–1.20 (1.23–1.20)	47.67–1.20 (1.23–1.20)
Observed reflections	1,000,823 (76,284)	1,262,949 (98,292)	1,112,726 (84,484)
Unique reflections	75,269 (5504)	76,342 (5611)	68,935 (5048)
Multiplicity	13.3 (13.9)	16.5 (17.5)	16.1 (16.7)
Completeness (%)	97.9 (98.2)	97.6 (97.9)	89.8 (90.2)
*R*_*merge*_	0.251 (0.963)	0.262 (1.004)	0.237 (0.936)
<*I/σI*>	6.59 (2.00)	6.71 (2.08)	7.18 (2.34)
CC_1/2_ (%)	99.2 (50.5)	99.0 (44.7)	99.1 (28.1)
**Refinement**			
Resolution (Å)	47.32–1.20	57.21–1.20	47.67–1.20
No. of reflections	75,213	76,038	68,168
R_work_/R_free_	0.153/0.170	0.167/0.182	0.158/0.196
<*B* > factors (Å^2^)			
Overall	10.40	11.13	10.74
Protein	8.32	9.05	8.54
Ligands/ions	9.82	10.54	8.99
Waters	24.15	24.87	25.24
R.m.s. deviations			
Bond lengths (Å)	0.015	0.011	0.015
Bond angles (°)	1.21	1.10	1.24

† Values in parenthesis are for the highest resolution shell. Data were truncated at 1.2 Å resolution to allow for a direct comparison of intensity and model statistics between different energy filter slit settings.

**Fig. 5:**
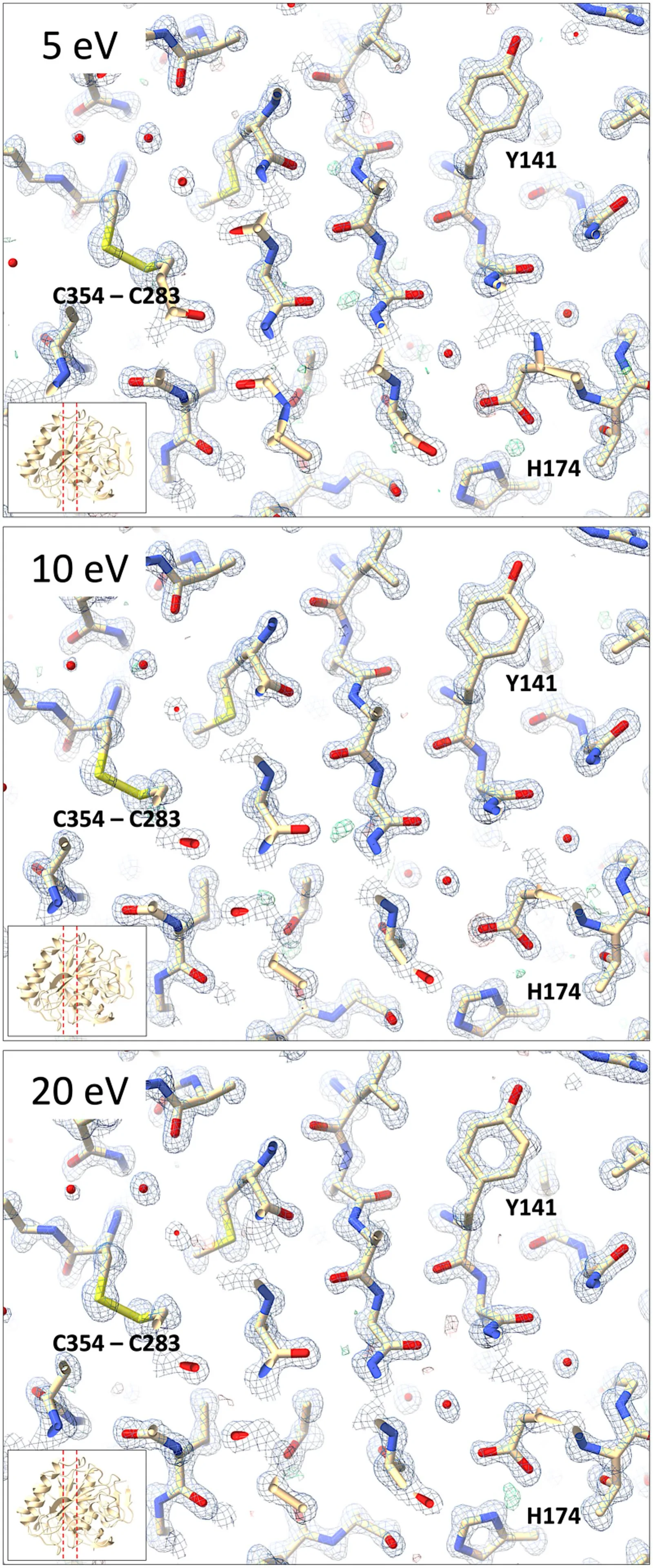
Electrostatic potential and difference maps for proteinase K from energy-filtered MicroED data using the 5, 10, and 20 eV slit settings. Data were truncated at 1.2 Å resolution. Electrostatic potential 2mFo-DFc maps are shown in blue and contoured at 2.2σ, difference mFo-DFc maps are shown in green and red for positive and negative density contoured at 3σ. No information for missing reflections was included for map calculations. (For interpretation of the references to colour in this figure legend, the reader is referred to the web version of this article.)

For a direct comparison in real space, structures were refined using the same starting model and the same refinement protocol. The 5 eV data show the lowest model *R*-factors with an *R*_work_/*R*_free_ of 0.153/0.170, as well as lower *B*-factors (Table 1). The *R*_free_ and *CC*_free_ values are better for the 5 eV data showing a less rapid falloff going towards the higher resolution shells compared to the 10 and 20 eV data that show a more similar trend (Fig. 4). Furthermore, correlation coefficients between the observed and calculated structure factor amplitudes show an improvement for the narrower slit widths (**Fig. S4**). A potential caveat is the overall completeness, which is lower for the 20 eV datasets at 89.8% (Table 1), which likely would not affect the intensity statistics too severely. However, the missing information could potentially affect structure refinement and map calculations. Upon visual inspection, the electrostatic potential maps did not show any noticeable differences between the different energy filter slit settings (Fig. 5).

## 4 Discussion

We assess the performance of a data collection strategy combining direct electron detection with energy filtering with different slit widths to separate the inelastic noise from the elastic diffraction signal. Previously, we demonstrated a clear improvement in SNR from unfiltered to energy-filtered MicroED data, leading to enhanced data quality and resolution (Clabbers et al., 2025). Here, we characterized the different energy filter slit widths settings by comparing diffraction stills and MicroED data (Fig. 3). To allow for a direct comparison, the data were all truncated at 1.2 Å resolution (Table 1). Even though the background noise is higher, the wider 20 eV slit settings showed the highest mean *I/σI* compared to the 5 and 10 eV data, which becomes even more pronounced for the lower resolution shells (Fig. 4). This can potentially be attributed to those events where electrons have undergone one or more inelastic scattering events after an initial elastic event. These electrons are still partially coherent and may contribute to increased counts near the Bragg spots. The wider 20 eV slit setting is less strict in separating out these partially coherent events. This could potentially improve the apparent contrast of the diffraction peaks relative to the background, but this does not necessarily yield more accurate intensities. In fact, the *CC*_1/2_ and*R*_work_/*R*_free_ are stronger for the 5 eV data, suggesting that the intensities that were measured with the narrower slit setting agree better internally and with the structural model (Table 1**,** Fig. 4). Additionally, there seems to be no significant loss of high-resolution information at 5 eV compared to the 10 and 20 eV slit data, which on average show more intense peaks (Fig. 3). A caveat of this comparison is that the 20 eV datasets were collected from a separate grid than the 5 and 10 eV datasets. Although all grids were prepared using the same protocol and multiple lamellae were analyzed for each condition, differences in crystal quality or lamella preparation may contribute to some variability between datasets.

These results indicate that narrower energy filter slit widths tend to perform better, suggesting there might be an advantage to exploring even narrower slit settings. Interestingly, despite showing the lowest background levels, diffraction stills and MicroED data collected using an even narrower 2 eV slit showed a significantly weaker signal compared to any of the other slit widths (Figs. S2-S3). On our system under the current protocol, the 2 eV slit is smaller than the selected area (SA) aperture, introducing artefacts from the slit interfering with the aperture and effectively limiting the field of view, thereby reducing the overall diffraction signal. In addition, the narrowest 2 eV slit setting may exclude part of the coherent diffraction signal, as the electron source on our system has an energy spread of *ΔE* ∼ 0.8 eV. These observations suggest that, in practice, an optimal slit width represents a balance between rejecting inelastically scattered electrons and preserving the useful diffraction signal. A slit that is too narrow may attenuate the transmitted beam, become more sensitive to alignment, or exclude electrons contributing to the coherent diffraction signal, whereas a wider slit admits more inelastic background. Our results indicate that, after careful alignment on our system, a 5 eV slit provides the best compromise between these effects, causing only minimal interference at the very edges of the SA aperture. Selecting the wider 10 and 20 eV slits did not obscure any of the apertures, making these potentially more robust to slight shifts of the energy filter slit alignment during prolonged operation. These wider slit settings show a substantial reduction in background noise compared to unfiltered data, but do not have as strong of a separation between signal and noise as observed for the 5 eV data (Fig. 1, Fig. 2). Altogether, the 5 eV energy filter slit width provides a substantial noise reduction while retaining a robust diffraction signal for reliable intensity measurements. Ultimately, the best protocol for optimal data quality differs per experiment and is not only a trade-off between the signal and the noise, but also depends on other factors including the flux density per frame, diffraction strength of the crystals, and the radiation sensitivity of the sample.

Our results illustrate the importance of optimizing the experimental setup and data collection strategies to allow for the best data quality. Energy filtering is an important part of enhancing data quality by selectively removing inelastically scattered electrons that contribute noise rather than a useful diffraction signal. Advances in better energy filters or more coherent electron sources can allow for even narrower slit widths, further decreasing the noise and improving the contrast. Detector improvements enabling higher frame rates and increased dynamic range for electron counting detectors reduce acquisition time, and allow for a higher fluence per frame without sacrificing accuracy (Hattne et al., 2023). Additionally, data collection strategies that combine even more small sweeps (Yonekura et al., 2015), or adopting a serial crystallography approach that uses snapshots of many individual crystals (Bücker et al., 2020), could provide a significant boost in attainable resolution. These approaches could increase redundancy and improve the accuracy of intensity measurements by better sampling reciprocal space, thus minimizing errors due to mechanical drift or crystal misalignment over longer data collection periods. By systematically refining data collection parameters such as energy filter slit width, fluence per frame, and total accumulated dose, MicroED experiments can be optimized for specific sample types, enhancing SNR and resolution in a controlled and reproducible way. Ultimately, optimal adjustment of the data collection parameters leads to better data quality, which informs better structural models.

## CRediT authorship contribution statement

**Max T.B. Clabbers:** Writing – review & editing, Writing – original draft, Visualization, Validation, Formal analysis, Data curation. **Johan Hattne:** Writing – review & editing, Writing – original draft, Formal analysis. **Michael W. Martynowycz:** Writing – original draft. **Tamir Gonen:** Writing – review & editing, Supervision, Project administration, Funding acquisition, Conceptualization.

## Declaration of competing interest

The authors declare that they have no known competing financial interests or personal relationships that could have appeared to influence the work reported in this paper.

## Data Availability

Coordinates and structure factors have been deposited in the Protein Data Bank (PDB) under accession codes 9N0F https://doi.org/10.2210/pdb9n0f/pdb (Proteinase K, 5 eV energy filter slit), 9N0G https://doi.org/10.2210/pdb9N0G/pdb(Proteinase K, 10 eV energy filter slit), 9N0H https://doi.org/10.2210/pdb9N0H/pdb (Proteinase K, 20 eV energy filter slit). EM maps have been deposited in the Electron Microscopy Data Bank (EMDB) under accession codes EMD-48783 https://www.emdataresource.org/EMD-48783 (Proteinase K, 5 eV energy filter slit), EMD-48784 https://www.emdataresource.org/EMD-48784 (Proteinase K, 10 eV energy filter slit), EMD-48785 https://www.emdataresource.org/EMD-48785 (Proteinase K, 20 eV energy filter slit).
