# Corrigendum to “Characterization of energy filtering slit widths for MicroED data collection” [J. Struct. Biol.: X 14 (2026) 100158]

**DOI:** 10.1016/j.yjsbx.2026.100160

**Published:** 2026-09-05

**Authors:** Max T.B. Clabbers, Johan Hattne, Michael W. Martynowycz, Tamir Gonen

**Affiliations:** a Howard Hughes Medical Institute, University of California, Los Angeles, CA 90095, United States; b Department of Biological Chemistry, University of California, Los Angeles, CA 90095, United States; c Department of Physiology, University of California, Los Angeles, CA 90095, United States

The authors regret that the Supplementary Information was missing from the original publication. This Corrigendum is to add the supplementary information to the publication.

The authors would like to apologise for any inconvenience caused.

