# Supplementary material for: Corrigendum to “Characterization of energy filtering slit widths for MicroED data collection” [J. Struct. Biol.: X 14 (2026) 100158]
Source: J Struct Biol X. 2026 Sep 5;14:100160. doi: 10.1016/j.yjsbx.2026.100160 (PMC13572022; doi:10.1016/j.yjsbx.2026.100160)
Supplement: Supplementary file 1 [file mmc1.pdf]

Supporting information for

Characterization of energy filtering slit widths for MicroED data collection

Max T.B. Clabbers[1,2], Johan Hattne[1,2], Michael W. Martynowycz[2] and Tamir Gonen[1,2,3,*]

[1]Howard Hughes Medical Institute, University of California, Los Angeles, CA 90095

[2]Department of Biological Chemistry, University of California, Los Angeles, CA 90095

[3]Department of Physiology, University of California, Los Angeles, CA 90095

*Corresponding author. E-mail address: tgonen@g.ucla.edu

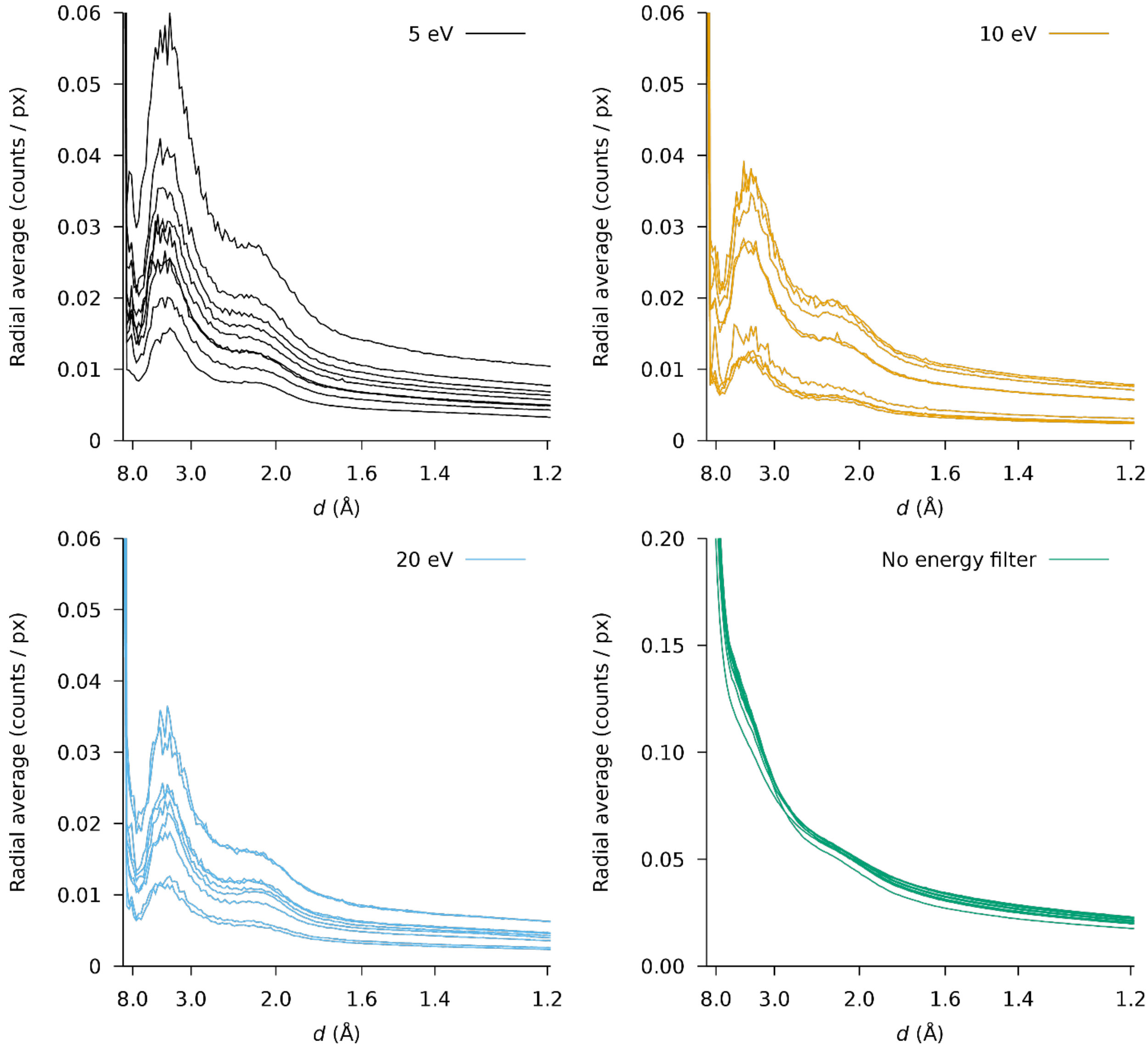

Figure S1 *Average number of counts per physical, unbinned pixel, as a function of resolution in the interval* $1.2 \leq d < \infty$ *Å. The curves correspond to single datasets, which are grouped by the slit width of the energy filter: 5 eV (black), 10 eV (orange), and 20 eV (blue). The green curve shows the radial average for datasets collected without the energy filter and has a different scale on the vertical axis. The variability between datasets is greater than the difference between the averages for each slit width.*

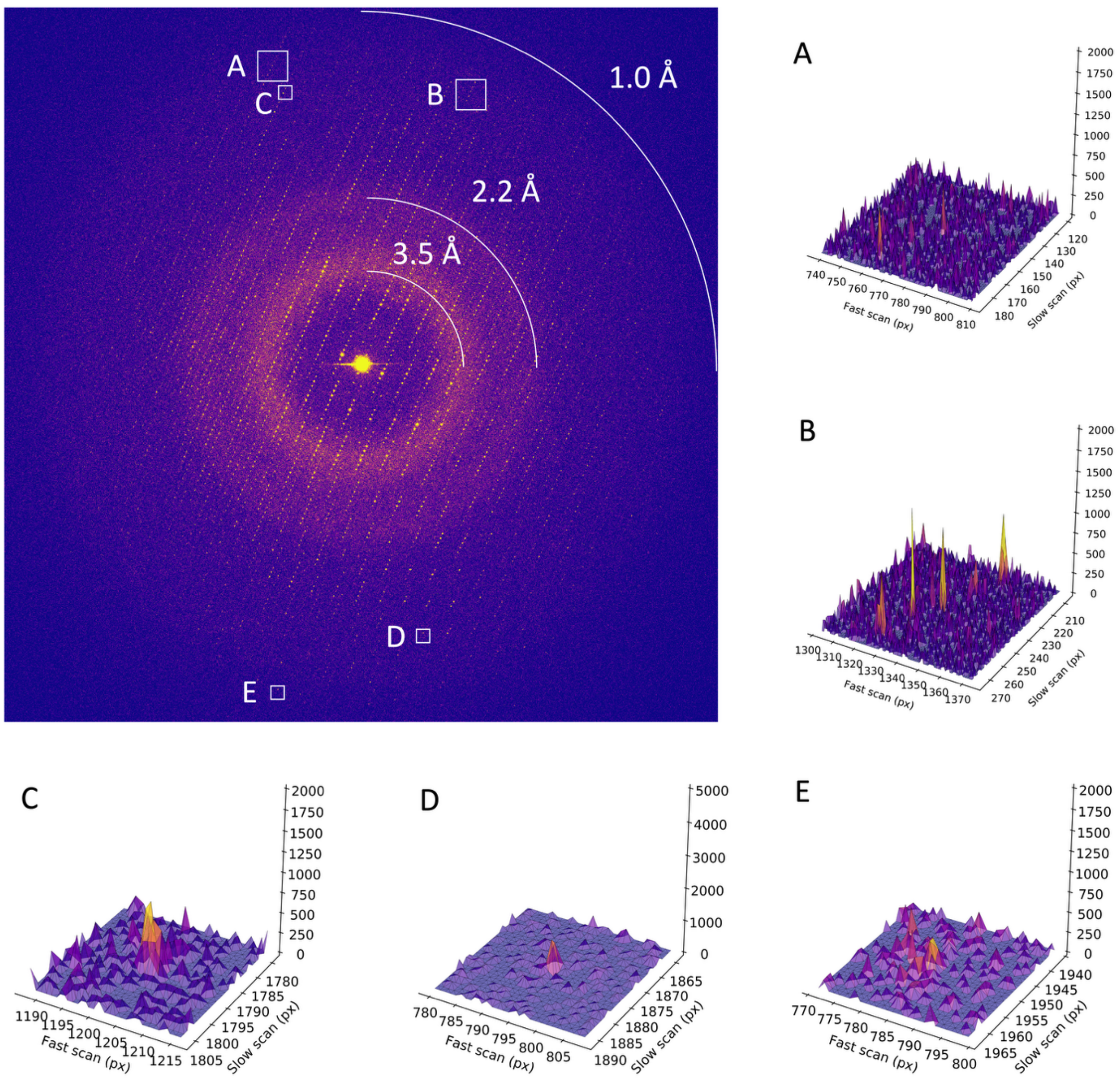

Figure S2 *Representative electron diffraction pattern taken with a 2 eV energy filter slit using a 10 s exposure at a total fluence of 0.02 e⁻/Å². Peak profiles are shown for each of the highlighted areas marked A-E. For reference, peak profiles are shown for Bragg spots at approximately **A**: 1.07 Å, **B**: 1.17 Å, **C**: 1.25 Å, **D**: 1.11 Å, and **E**: 1.02 Å resolution.*

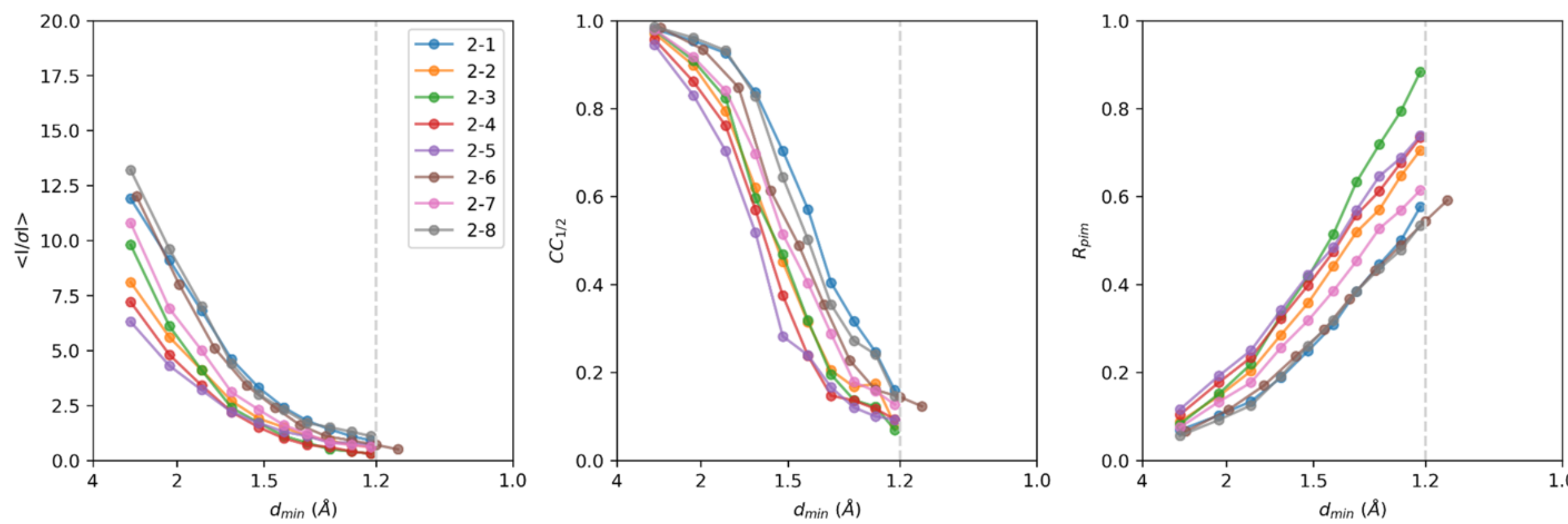

Figure S3 *Intensity statistics for individual datasets collected using a 2 eV energy filter slit. The crystallographic quality indicators mean I/σI, $CC_{1/2}$, and R_{pim} are plotted as a function of the resolution.*

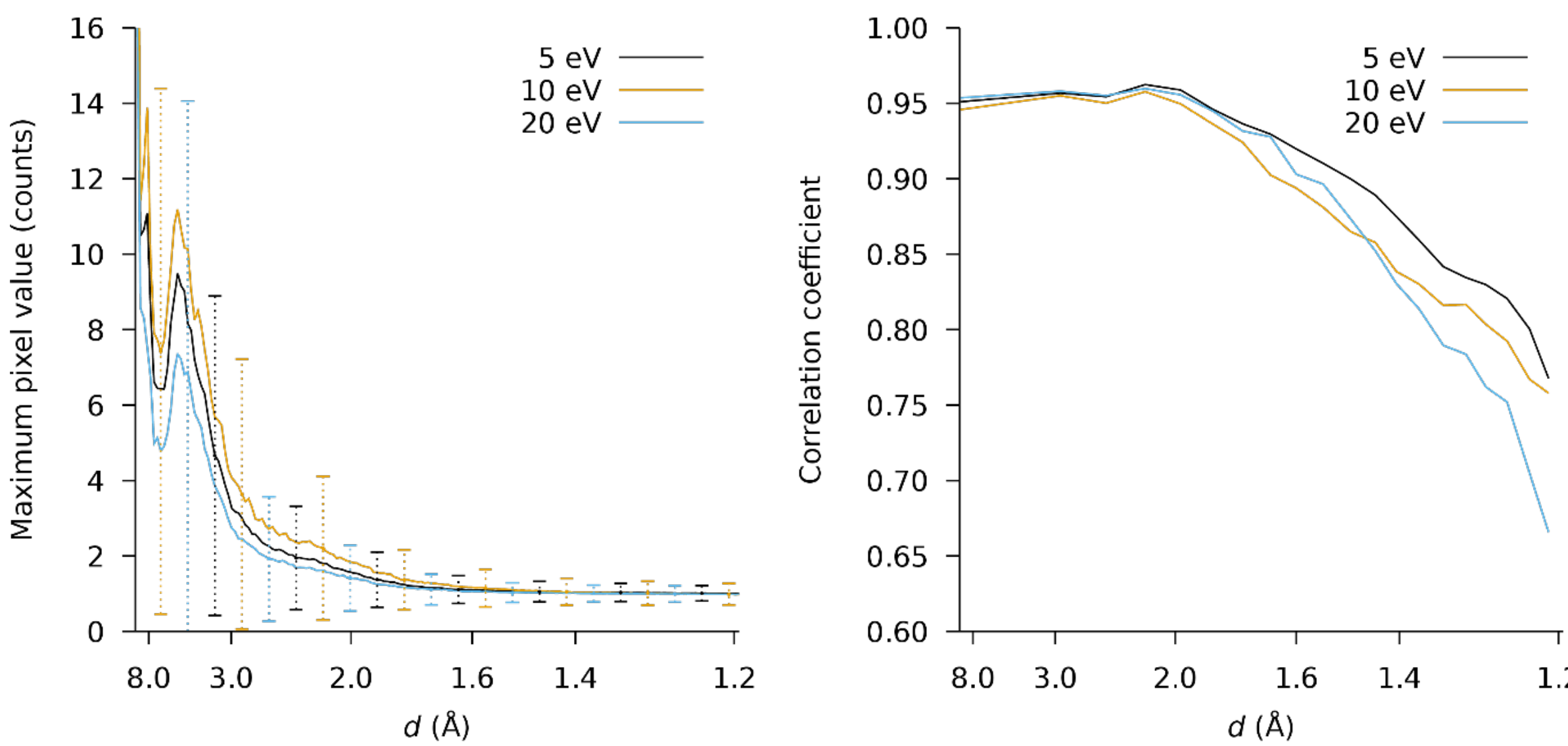

Figure S4 *Average pixel value of the brightest integrated pixel in the resolution interval* $1.2 \leq d < \infty$ *Å for the three slit widths of the energy filter (left) and correlation between observed and calculated structure factor amplitudes for the models presented in the main text (right). The dotted error bars indicate one standard deviation. We note that a narrower slit width does not unconditionally imply lower peak pixel values in the integrated spots.*
